# Authentic Leadership and Employees’ Innovative Behaviour: A Multilevel Investigation in Three Countries

**DOI:** 10.3390/ijerph16214201

**Published:** 2019-10-30

**Authors:** Mariola Laguna, Karolina Walachowska, Marjan J. Gorgievski-Duijvesteijn, Juan A. Moriano

**Affiliations:** 1 Institute of Psychology, The John Paul II Catholic University of Lublin, 20-950 Lublin, Poland; walachowska.karolina@gmail.com; 2 Department of Work and Organisational Psychology, Erasmus University Rotterdam, 3000 DR Rotterdam, The Netherlands; gorgievski@essb.eur.nl; 3 Department of Social and Organisational Psychology, The National Distance Education University (UNED), 28040 Madrid, Spain; jamoriano@psi.uned.es

**Keywords:** leadership, authentic leadership, innovation, innovative behaviour, personal initiative, work engagement, business owners, entrepreneurship, multilevel analysis

## Abstract

The innovativeness of individual employees is a vital source of competitive advantage of firms, contributing to societal development. Therefore, the aim of this multilevel study was to examine how entrepreneurial firm owners’ authentic leadership relates to their employees’ innovative behaviour. Our conceptual model postulates that the relationship between business owners’ authentic leadership (as perceived by their employees) and their employees’ innovative behaviour is mediated by employees’ personal initiative and their work engagement. Hypotheses derived from this model were tested on data collected from 711 employees working in 85 small firms from three European countries: the Netherlands, Poland, and Spain. The results of the multilevel modelling confirmed our model, showing that when business owners are perceived as more authentic leaders, their employees show higher personal initiative and are more engaged at work and, in turn, identify more innovative solutions to be implemented in the organization. A cross-national difference was observed: employees from Spain (in comparison to Dutch and Polish employees) reported engaging less frequently in innovative behaviour. These research findings suggest that the innovative behaviour of employees can be boosted through leadership training, improving the quality of relationships between leaders and subordinates, and strengthening employees’ personal initiative and work engagement.

## 1. Introduction

In the present era, the implementation of innovative ideas in organizations is crucial, both for the long-term survival of a firm [1] and for the development of new solutions that protect the environment and the well-being of societies [2]. Studies on innovations in general and on ecological innovations specifically tend to focus on external influences (such as policy making and market opportunities), but usually ignore the processes within the company—the mechanisms operating at the micro-level which stimulate innovative behaviour [3,4,5]. The innovative behaviour of employees, i.e., creation and implementation of new solutions in the workplace as manifested in everyday activities, is an important micro foundation of the innovativeness of the whole firm [6]. This especially prominent in small firms, which provide the majority of private employment [7] and do not have special innovation departments, and thus the innovative behaviour of their employees is major driver of the organization’s innovation [6]. In addition, the entrepreneurial owner who operates the firm is usually also the manager and supervisor of his or her employees. Thus, his or her leadership behaviours are of vital importance for creating a working environment that fosters employees’ innovative behaviour [8]. Taking into account that entrepreneurs in small firms perform multiple roles and are usually direct managers of their employees, we treat them as leaders of these organizations, as proposed in other research [9].

Leadership may cover a variety of conducts, and the influence of leader’s characteristics on employee behaviour has become an emerging research area [10]. This study builds on the theory of authentic leadership [11]. Today, as the disreputable conduct of some company managers has been unveiled, it is significant to direct attention to the leaders’ behaviours in terms of displaying sincerity and authenticity in work relationships rather than manipulation or work towards hidden purposes. Authentic leadership theory defines the authentic leader as being characterized by high self-awareness, balanced processing of information, relational transparency and internalized moral perspective, and acting in accordance with their inner thoughts and feelings [11,12]. Research has explored the influence of authentic leadership on a variety of work attitudes [12]. However, leadership scholars have identified a lack of research addressing underlying mechanisms that explain how leaders influence important processes in organizations, including stimulation of innovativeness. Leadership scholars have also noted that more studies are needed that investigate the effects of leadership behaviours at different levels of analysis, such as linking leaders’ characteristics to the attitudes and behaviours of their employees within teams or organisations [13].

Filling these gaps in the literature, the present study investigates underlying psychological mechanisms linking entrepreneurial owners’ authentic leadership and employee innovative behaviour. We propose and test a multilevel model which postulates that the more authentic the leader is perceived to be, the more innovative behaviour his or her followers show. The evidence posits that authentic leadership enhances employees’ feelings of involvement and commitment [12]. This suggests that personal motivational variables related to involvement and engagement may play a role in transferring leader’s attitudes into employees’ behaviours. Based on this, we postulate that the relationship between the authentic leadership displayed by the entrepreneurial owner and his or her employees’ innovative behaviour is mediated by employees’ work engagement [14] and their personal initiative [15]. If the leader exhibits authenticity, his or her followers are inspired by their supervisor, they are likely to become more engaged with their assignments, and are more willing to go beyond their job description. High work engagement and personal initiative are expected to result in increased innovative behaviour, as they equip employees with energy and motivation [14,15], and enable them to introduce novel practices in the workplace.

Our study contributes to the innovation and leadership literature in several ways. Firstly, applying a multilevel study design allows us to analyse the relations between the leadership style of entrepreneurial firm owners (representing a higher level of analysis—Level 2) and their employees’ work behaviour (representing a lower level, i.e., individual-level—Level 1). Secondly, this study is among the first to test the explanatory mechanisms linking employee innovation and authentic leadership attitudes and behaviours through individual-level, psychological variables. Thirdly, we will analyse data of employees from three European countries. Hence, the study provides preliminary evidence for the extent to which results generalize across different cultures as well as possible cross-country differences.

### 1.1. Authentic Leadership and the Innovative Behaviour of Employees

Innovation is the creation of valuable and useful new products, services or production processes in the organizational context [16]. It has been proven that both large corporations and small companies develop and prosper in the long term due to the innovative potential of employees [17]. In small organizations, the business owner usually also manages the company. Therefore, his or her attitudes and leadership behaviours are especially influential. The importance of managers’ support for employees’ innovative behaviour has been confirmed in earlier studies [18]. In a meta-analysis concerning predictors of the innovation of organizational members, Hammond and colleagues [19] revealed that immediate supervisors are able to spur innovation in subordinates.

According to authentic leadership theory, authentic leaders are able to stimulate innovation by encouraging their followers to be more daring and imaginative [20]. High relational transparency enables managers to openly show support and express that they value the capacities of subordinates and want them to perform well [21]. Therefore, such leaders build personal capital in their employees, who then may be more willing to put their ideas to use and find different pathways for solving problems. Moreover, authentic leaders have a strong inclination to promote diversity in ideas and perspectives among their followers, as they are more tolerant of ambiguity and open to experience [20]. Consequently, subordinates will feel freer to challenge the established patterns in the workplace and view the leader as a credible source of input and feedback over time [22]. Additionally, authentic leaders tend to be more self-confident and not afraid of taking risks and experimenting themselves [23]. They can be expected to stimulate employees’ innovative behaviour through modelling innovative behaviour besides creating an atmosphere that supports originality [20]. Indeed, studies have provided evidence for the relationship between authentic leadership and innovation. Zhou and colleagues [21] tested the relationship between authentic leadership and employee innovation, taking into account the mediating effect of employees’ positive emotions. These results showed that leaders who are perceived as more authentic are able to evoke positive emotions (courage and enthusiasm), which in turn leads to proposing innovative solutions at work. Müceldili and colleagues [24] showed a positive relation between authentic leadership and employee innovative behaviour, which was mediated by employee creativity. Other studies offered indirect evidence, showing that perceived authentic leadership was related to employee creativity [25,26] or an innovative group climate and information sharing in teams [27]. Studies additionally showed that a positive relationship between authentic leadership and employee creativity is mediated by employee psychological resources and attitudes at work [20]. Most studies did not take into account the multilevel structure of organizations, which is why these findings await further replication using a multilevel approach, as multilevel and single-level relationships do not necessarily correspond [28].

Authentic leadership is not easy to study. Previous research has shown that there is a discrepancy between how authentic leaders view themselves and how they are perceived by their subordinates [29]. This finding presents an interesting conundrum, as it is contradictory to authenticity theory, which posits that self-awareness, knowing one’s ‘true self’ and behaving in accordance with it, will automatically be communicated to followers who will experience the leader as authentic [30]. Furthermore, the interior states of managers are not always easy to notice by observers, yet “on a practical level, followers’ perceptions of the authenticity of a leader are as important to consider as are the actual thoughts and actions of the leader being perceived” [31] (p. 254). In line with this, a multilevel study conducted by Černe et al. [32] demonstrated that perceived authentic leadership had a positive effect on employee creativity and team innovativeness, whereas team leaders’ self-ascribed authentic leadership did not show such effects. The authors concluded that authentic behaviour clearly demonstrated to others, rather than leaders’ self-perceived authentic leadership, is more likely to lead to positive outcomes [32]. On the basis of this reasoning, evaluations of leader authenticity provided by employees were deemed a more reliable source of information, especially when these perceptions are intersubjective, meaning they are based on the evaluations of multiple observers. Similarly, also the assessment of employees’ innovative behaviour is not easy and self-assessment was recommended as a good option [33]. Employees’ innovative behaviour self-ratings have been found to be significantly correlated with their supervisors’ ratings [34,35] and with objective measures of invention disclosures [18]. Thus, acknowledging limitations of self-reports [36] but taking into account that data from other sources do not warrant a more objective measurement of individual innovation [33], we investigate self-evaluations of employees’ innovative behaviour. Therefore, the presented multilevel study investigates the authentic leadership of entrepreneurial firm owners as perceived by their employees (intersubjective) and examines the relationship between a leader and his/her followers’ innovative behaviour. Based on the theories and research presented above, we propose the following:

**H1:**  Authentic leadership is positively related to the innovative behaviour of employees.

### 1.2. Mediating Role of Personal Initiative and Work Engagement

**Figure 1:**
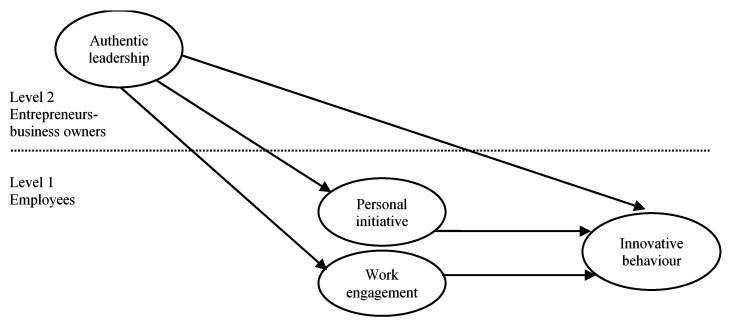
Conceptual model of relationships between variables.

In the present study, we focus on motivational variables that may explain the relationship between leadership and innovation at work. Based on an integration of innovation theories [37] and authentic leadership theory [11,12] two mechanisms were identified that may serve as mediators between authentic leadership and the innovative behaviour of employees [18], namely personal initiative and work engagement. Our research model is depicted in Figure 1.

Personal initiative [15] is defined as active and initiative-taking behaviour that goes beyond the formal requirements at work and is aimed at accomplishing both organizational and personal goals. Personal initiative is described as self-starting and being proactive, and the person who takes initiative is persistent in overcoming barriers [38]. It is proposed that employees who display initiative at work are keen on exploring opportunities that others do not consider [39]. As suggested by authentic leadership theory [11], authentic leaders who refer to their true self, admit and learn from mistakes, and focus on development would stimulate employees to initiate their own growth. Through the process of positive modelling, positive feedback and consequent followership development, employees would realize that they can achieve more than they previously thought, which should improve the quality of their activities [40].

As authentic leadership is a fairly new concept in the field of organizational psychology, its relationship to personal initiative has not yet been thoroughly tested empirically. Nevertheless, the literature offers some empirical evidence for the links between other conceptualizations of leadership and personal initiative or proactivity, a concept that is treated as one of components of personal initiative [38]. A recent meta-analysis [41] revealed that proactive performance by subordinates is associated with three conceptualizations of leadership, namely contingent reward leadership, leader–member exchange, and transformational leadership. Hence, it seems plausible that authentic leadership, which is both relationship-oriented and entails follower development, may also relate to the proactive behaviours of employees. Upholding this view, research findings on leader support show that leaders’ availability, encouragement, and non-interference predict employee proactive work behaviour [42].

We can also expect that employees’ personal initiative is positively related to their innovative behaviour. The authors of the personal initiative conception [15,43] proposed that such an initiative is valuable for the innovation process. Personal initiative is characterized by a self-starting approach and exerting effort to overcome obstacles, which is necessary for introducing ideas into an organization. In addition, people with a high personal initiative are proactive, find unusual ways of tackling problems, and are future oriented, which can make them more mindful of perceiving opportunities, finding new applications for products and developing creative ideas [44]. This assumption was confirmed by Frese et al. [45], who found personal initiative to be related to the number of improvement suggestions, to a problem-focused coping strategy, and to plan execution [15]. Other studies indicated that personal initiative is positively related to small firms’ success [46], addressing challenges and organizational-level performance [38]. Furthermore, there are a few reports that proved the relationship between personal initiative and innovation-related behaviour. Specifically, personal initiative predicts creativity [47], has a positive impact on work-unit innovativeness over time [48,49], and is associated with idea generation [44] and idea implementation [50], both of which are components of innovative behaviour at work. Therefore, theories of authentic leadership and personal initiative [11,15] and previous research findings provide reasons to expect the following:

**H2:**  Personal initiative mediates the relation between authentic leadership and the innovative behaviour of employees.

The second mechanism investigated in the present study proposes that work engagement is a mediator between authentic leadership and the innovative behaviour of employees. Work engagement is a positive and fulfilling affective-cognitive state related to work, which is characterized by vigour, dedication, and absorption in the activity [14]. Stander et al. [51] suggested that when leaders show authenticity, their employees are more optimistic and have more trust in the organization, which ultimately leads to higher work engagement. Research findings indeed revealed that authentic leadership increases work engagement among employees and their job performance [52,53]. Furthermore, there is a positive relationship between authentic leadership, as rated by employees, and employees’ work engagement [54].

In our model, we also postulate that work engagement is positively related to innovative behaviour. It is proposed that engaged employees are more productive and creative, as they often experience positive emotions, create their own job and personal resources, and are able to transfer their engaged attitude to others [55]. As introducing innovations involves putting in considerable effort, individuals have to be dedicated to their job to go the extra mile and come up with new ideas for the benefit of the organization. Moreover, to make an idea reality, employees must be energetic, resilient, and immersed in the task [14]. High work engagement may be important in exploring unconventional options and implementing original ideas in the organization [56]. In support of these theoretical assumptions, the evidence in the literature revealed that work engagement is positively related to a positive affect at work [57,58], to innovative work behaviour [59], and innovativeness [60,61].

There is some evidence concerning the mediating role of work engagement. Agarwal and colleagues [62] reported that high quality of social exchanges between employees and supervisors influence the work engagement of employees, which is then associated positively with employees’ innovative behaviour. This result is consistent with the finding that leadership is positively related to innovation through a mediating effect of work engagement in a study of doctors and nurses [63]. Based on the theoretical and empirical evidence presented above, the following hypothesis is posited:

**H3:**  Work engagement mediates the relation between authentic leadership and the innovative behaviour of employees.

### 1.3. Importance of National Culture

Management activities may depend on the national culture in which a firm operates [64,65]. Culture influences how leaders are perceived, which is why scholars express the need to examine how leadership is viewed in different cultures and situations [66]. Additionally, innovation and its mechanisms are, to some degree, culture-specific [67]. For example, a study in Jordan showed a relationship between transformational leadership and a company’s process and product innovation, but no such relationship was found for authentic leadership [68]. These findings led the authors to conclude that this relationship might be culture-specific. However, previous studies rarely considered a cross-cultural perspective, and empirical evidence on the relationships between authentic leadership and innovative behaviour that takes culture into account is lacking. Our study aims to contribute to a better understanding of possible cross-national differences by testing the hypotheses in firms from three different European countries. Thanks to established cooperation between researchers, we gathered data from the Netherlands, Poland, and Spain, which differ significantly in life conditions and cultural dimensions [64]. For example, these three countries differ in individualism and power distance [64,67], cultural dimensions that are important for innovation [69]. Moreover, different trends have been observed in the innovation levels of these countries: since 2012, innovation performance in the Netherlands and Poland has been growing, while in Spain, it has started to decrease [70]. Therefore, including these countries is particularly suited for gaining insight in possible cross-cultural similarities and differences in mechanisms linking business owners’ authentic leadership to employee innovative behaviour.

## 2. Materials and Methods

### 2.1. Procedure

Using formal and informal networks of small enterprises, entrepreneur who were owners of firms employing from 10 to 50 people were invited to take part in the study. The entrepreneurial owner of each firm fulfilled (jointly) four criteria, being (1) founder or successor of a firm, (2) business owner, and (3) manager of a firm that (4) had existed on the market for more than one year. If they agreed to include their firms into the study, we invited their employees to complete questionnaires. The only criterion used for the selection of employees was having a work agreement with the company taking part in the study.

In Poland and Spain, having completed the questionnaires in their native languages, the participants put the measures in envelopes, which guaranteed confidentiality and the anonymity of the data. In the Netherlands, participants were provided with access to electronic platform and filled in electronic versions of all the questionnaires. All the respondents participated voluntarily and did not receive any compensation for their contribution.

### 2.2. Participants

Participants were employees of small firms. In total, 711 employees from three countries took part in the study (174 from the Netherlands, 344 from Poland, and 193 from Spain); 57% of them were women, the mean age was 37 (*SD* = 10.66), and their mean duration of working in the organization was 6.35 years (range from 1 to 39 years, *SD* = 6.22). Of the sample, 71%were full time employees, 16% had partial employment, and 13% had other types of work agreements.

Altogether, the number of firms from the three counties was 85 (22 from the Netherlands, 37 from Poland, and 26 from Spain). Sixty-five of the entrepreneurs-firm managers were male. Their firms operated in services and trade (44%), production/manufacturing (30%), construction (11%), and other branches of industry (15%). The mean age of the firms was 22 years (range from 1 to 128 years, *SD* = 26.64 years).

### 2.3. Measures

#### 2.3.1. Authentic Leadership

National versions of the Authentic Leadership Questionnaire [22] were used. An example item is “*My leader encourages everyone to speak their mind*”. The questionnaire is comprised of four subscales assessing leader self-awareness, relational transparency, internalized moral perspective, and balanced processing. In the process of the national adaptations of the measure [71,72], three items were deleted from the original version of the scale (items 4, 5, and 7). Responses were given on a five-point scale from 0—*not at all* to 4—*frequently, if not always*. The score of authentic leadership is calculated as the total score of all the items; the scores range from 0 to 52. The Cronbach’s α in the present study was 0.94. Using confirmatory factor analysis (CFA) on the data from the three countries, the four-factor solution was confirmed (χ^2^_(59)_ = 330.617, *p* < 0.001, Comparative Fit Index (CFI) = 0.961, Root Mean Square Error of Approximation (RMSEA) = 0.074, Standardized Root Mean Square Residual (SRMR) = 0.030).

#### 2.3.2. Personal Initiative

Three national versions of the 7-item Personal Initiative Scale [15] were used (example item: “*Whenever something goes wrong, I search for a solution immediately*”). The scale was translated into national languages using collaborative-iterative translation [73]. Responses were given on a five-point scale, ranging from 1—*not true at all* to 5—*absolutely true*. The total scores range from 7 to 35. The single-factor CFA model for personal initiative fit the data quite well, except for the RMSEA value (χ^2^_(14)_ = 169.925, *p < 0*.001, CFI = 0.943, RMSEA = 0.114, SRMR = 0.040). Cronbach’s α was 0.89.

#### 2.3.3. Work Engagement

National versions of the 9-item Utrecht Work Engagement Scale [14,74,75] were used. The three dimensions of work engagement are: vigour, dedication, and absorption, which form a global score of work engagement, ranging from 0 to 54 (example item: “*At work, I feel bursting with energy*”). Responses were given on a seven-point scale from 0—*never* to 6—*always*. The Cronbach’s α was 0.92. The three-factor CFA model for work engagement fit the data quite well, except for the RMSEA value (χ^2^_(27)_ = 301.085, *p* < 0.001, CFI = 0.947, RMSEA = 0.109, SRMR = 0.036).

#### 2.3.4. Innovative Behaviour

National versions of the Innovative Behaviour Questionnaire [18,76] were used. Where the national versions were unavailable, the scale was translated using collaborative-iterative translation [73]. The measure consists of six items (example of item: “*I search out new technologies, processes, techniques, and/or product ideas*”). Responses range from 1—*never* to 5—*very often*, and a total score from 6 to 30. The Cronbach’s α was 0.92. Confirmatory factor analysis of the single-factor model showed acceptable fit to the data (χ^2^_(6)_ = 35.912, *p* < 0.001, CFI = 0.992, RMSEA = 0.077, SRMR = 0.014).

### 2.4. Data Analysis Strategy

As the first step, the measurement invariance of each scale across the samples from the three countries was investigated. Using Multigroup Confirmatory Factor Analysis (MGCFA, [77,78]) in AMOS 23, we first examined the configural invariance by estimating the same model in all three groups without cross-group constraints. Next, we proceeded with testing more rigorous conditions that required equivalent factor loadings (metric invariance, [77]). Then, we constrained equivalent intercepts, called scalar invariance [77]. If the measure does not achieve full invariance, it is appropriate to test its partial invariance, allowing some parameters to vary across samples [78]. To test the differences between increasingly restricted nested models, we calculated Δχ^2^ and ΔCFI. An absolute difference in CFI that is less than 0.01 would indicate measurement invariance [79].

As our data have a multilevel structure with employees on Level 1 and entrepreneurs on Level 2, we applied hierarchical multilevel modelling [80] using HLM 7 software [81], both in these preliminary analyses testing the potential effects of sex, age, and country on innovative behaviour and in the hypothesis testing. Following the recommendations [80], in all the analyses, continuous variables from Level 1 were group mean centred, and continuous variables from Level 2 were standardized and uncentred. For all the multilevel analyses, unstandardized gamma coefficients (γ) are reported.

To test our hypotheses, authentic leadership at the entrepreneur’s level was calculated as a mean value of all the scores provided by employees within each firm (aggregated scores at Level 2). In a series of the multilevel equations, employee innovative behaviour (dependent variable measured at Level 1) was explained by predictors from Level 2 (authentic leadership and country effect) and Level 1 (personal initiative and work engagement). In Model 1, the role of authentic leadership was analysed. Model 2 verified authentic leadership and personal initiative in explaining innovative behaviour. Model 3 tested the role of authentic leadership and work engagement in explaining innovative behaviour. Model 4 included all the independent variables together: authentic leadership, personal initiative and work engagement in predicting employee innovative behaviour.

Our hypotheses postulate that the effects of authentic leadership on innovative behaviour are mediated by personal initiative and work engagement. To test these hypotheses, in the next multilevel analyses, personal initiative and work engagement were treated as dependent variables and authentic leadership as a predictor, controlling for the country effect. We used PRODCLIN [82] to test for the statistical significance of the mediation effect. This program allows testing of indirect effects by computing confidence intervals (CI) for the values resulting from multilevel analysis. When the CI does not include zero, a significant mediation effect is confirmed.

## 3. Results

### 3.1. Measurement Equivalence

We applied MGCFA to examine the measurement invariance of all the scales used in the study across the samples of employees from the three countries [77,78]. For each scale, the configural unrestricted model showed a good fit to the data (Table 1; Model 1 for each measure). Next, more stringent equality constraints were imposed across the three countries for each scale. For the authentic leadership measure, partial scalar invariance was achieved (freeing factor loadings of item 2). For the personal initiative and work engagement measures, full metric invariance was confirmed, and for the innovative behaviour measure, full scalar invariance was established between countries. These results allow meaningful analyses of the relationships between the constructs across the countries.

### 3.2. Variance Distribution and Control Variables

Table 2 shows the means, standard deviations and correlations between the study variables. Positive and statistically significant correlations between all the variables provide initial confirmation of our hypotheses.

The dataset consisted of two hierarchically nested levels: 711 employees (Level 1) working in 85 organizations led by different entrepreneurs (Level 2). To check for variance distribution and analyse whether demographic variables and country effects are statistically significant predictors of the innovative behaviour of employees, we applied multilevel modelling [68]. First, an unconditional model (without any predictor; Model 0) showed variance in innovative behaviour (dependent variable) on both levels: 17.59 on the individual level and 6.85 on the entrepreneur level. This finding indicates that individual employees differ in terms of their innovative behaviour, and firms differ in how innovative their employees are. This finding confirmed our choice of multilevel modelling as relevant for examining the relationships between the variables.

Next, we tested for demographic variables as potential predictors of employee innovative behaviour. Neither employee age (γ = 0.02, *SE* = 0.02, *p* = 0.351) nor sex (γ = −0.82, *SE* = 0.45, *p* = 0.056) were found to be a significant predictor of their innovative behaviour. Consequently, following a recommendation to keep only statistically significant predictors in a multilevel model (forward stepping [67]), these variables were not taken into account in further analyses. Nevertheless, we observed a significant country effect (γ = −0.93, *SE* = 0.39, *p* = 0.02). Further analyses showed that this effect refers to a contrast between Spain and the other countries: being an employee from Spain (contrast coded Spain = 1, other countries = 0) decreases the level of innovative behaviour (γ = −1.63, *SE* = 0.70, *p* = 0.02). Such effects were insignificant for the other countries (as contrast coded), the Netherlands (γ = 0.90, *p* = 0.16), and Poland (γ = 0.68, *p* = 0.30). Therefore, in all subsequent analyses, we controlled for country effect, including Spain as a contrast coded variable at Level 2 in each equation.

### 3.3. Hypotheses Testing

We used hierarchical multilevel modelling to test our hypotheses [81]. In Model 1 (Table 3), we examined the relationship between perceived authentic leadership (Level 2) and employee innovative behaviour (Level 1). The positive and statistically significant coefficient (γ = 1.09, *p* = 0.001) showed that the more authentic the entrepreneur is, the higher the innovative behaviour of his or her employees. Hypothesis H1 was thus confirmed. In this model, the country effect was also statistically significant (γ = −2.02, *p* = 0.002).

Next, we separately tested for each of two proposed mediators. Model 2 involved testing the effect of employee personal initiative on employees’ innovative behaviour, controlling for perceived authentic leadership and country effects. The coefficient was positive and statistically significant (γ = 0.47, *p* = 0.001). In Model 3, the relationship between employee work engagement and innovative behaviour was tested in the same manner. The coefficient of work engagement was positive and statistically significant (γ = 0.23, *p* = 0.001). Therefore, both personal initiative and employee work engagement are positively related to innovative behaviour.

In Model 4, we tested for the role of authentic leadership, personal initiative and work engagement in explaining the variance in employee innovative behaviour. It was found that each of these variables predicts employee innovative behaviour when all of them are included in the multilevel equation. The coefficients were positive and statistically significant for authentic leadership (γ = 1.07, *p* = 0.001), personal initiative (γ = 0.38, *p* = 0.001), and work engagement (γ = 0.13, *p* = 0.001). The country effect was also found to be statistically significant (γ = −0.04, *p* = 0.002).

Testing the mediation hypotheses, we first performed separate multilevel analyses for personal initiative and work engagement. In the unconditional model, personal initiative was found to vary more among employees (Level 1 variance = 18.80) than among firms led by different entrepreneurs (Level 2 variance = 4.05), i.e., at both levels. The country effect in predicting personal initiative was not statistically significant (γ = −0.34, SE = 0.53, *p* = 0.52). Authentic leadership was found to be a statistically significant and positive predictor (γ = 0.73, SE = 0.25, *p* = 0.004), meaning that if the entrepreneur was seen as more authentic by employees, they displayed higher personal initiative. The PRODCLIN analysis provided statistical support for the idea that personal initiative is a mediator between authentic leadership and the innovative behaviour of employees (95% CI [0.09, 0.49]). Employees managed by more authentic entrepreneurs take more initiative at work and thus show a higher innovative behaviour. Hypothesis H2 was therefore confirmed.

Similarly, the variance in work engagement between employees of a given firm was higher (Level 1 variance = 59.53) than the variance between firms lead by entrepreneurs (Level 2 variance = 18.45). The effect of the country in predicting work engagement was statistically significant (γ = −2.78, *p* = 0.019). Authentic leadership was positively related to work engagement (γ = 1.79, SE = 0.52, *p* = 0.001); the more authentic an entrepreneur was, the higher was their employees’ work engagement. The results of the PRODCLIN analysis (95% CI [0.08, 0.42]) confirmed that work engagement serves as a mediator between authentic leadership and the innovative behaviour of employees. Entrepreneur’s authentic leadership style stimulates employees’ work engagement, which in turn is reflected in their innovative behaviour displayed in the workplace. This finding supported Hypothesis H3.

## 4. Discussion

Innovation, as the development of new solutions, brings not only economic gains for firms, but also introduces changes which contribute to the well-being of societies and to the protection of the environment [2]. Therefore better understanding what drives implementation of innovative ideas, especially in small firms providing the majority of private employment [7], is of high societal importance. In our study we looked at the relationships between entrepreneur-owner’s leadership style and their employees’ innovative behaviour. Answering the call for examining the effects of leadership behaviour at different levels of analysis as well as for explaining mechanisms responsible for these effects [13], our study investigated the multilevel relationships between perceived authentic leadership and the innovative behaviour of employees, as captured by their self-evaluations. First, we demonstrated that the more authentic the entrepreneur is to be perceived as a leader, the higher the innovative behaviour of his or her employees. Second, we offered insight into the mechanisms that underlay these relationships, namely, that authentic leadership predicts personal initiative and the work engagement of employees, and these variables in turn predict employees’ innovative behaviour. Our results show that employee personal initiative and work engagement are mediators of the relationship between the authentic leadership of entrepreneur-firm managers and the innovative behaviour of their employees. Entrepreneurs leading small firms, when being authentic and perceived as authentic at work, stimulate personal initiative and higher work engagement in their employees, encouraging them to undertake innovative behaviour at work. Such leaders can encourage the free exchange of ideas and mutual support among their employees, which is conducive to innovation [83].

In addition to confirming the previous findings linking authentic leadership with positive outcomes in employees [84], our multilevel study provides empirical support for the theory of authentic leadership [11], confirming that authentic leaders encourage employee innovation. Furthermore, contact with authentic leaders results in the increased meaningfulness and significance of work [83], which may explain why employees are more engaged at work and take greater initiative, as shown by our results. Previous research has provided evidence that authentic leadership relates to innovation through positive emotions [21], psychological capital [26], affective commitment, and job resourcefulness [25]. We extend this line of research and show evidence for personal initiative and work engagement as two individual-level variables through which the effects of authentic leader attitudes on followers are transferred.

Conducting the research in three countries, which are different in terms of national cultural dimensions [67], allowed us to control for culture effects. Importantly, even though the countries differ substantially [64,70], the model largely holds across different socio-cultural environments. However, our results showed that being an employee from Spain decreases innovative behaviour in comparison to Dutch and Polish employees. This finding is in line with findings showing that Spain’s innovation performance has dropped in recent years, whereas in Poland and the Netherlands, it has improved [70]. One of the possible reasons for this may be rather high in-group collectivism in Spain [67], which is characterized by the strong interdependence of group members as well as rigid group structures and hierarchies, which can curb the exchange of ideas, lower the readiness to change, and increase the danger of group thinking [85]. Individualistic cultures value freedom more than collectivistic cultures, and because of this, employees may have more opportunities to try out new things at work [86].

### 4.1. Limitations and Future Directions of Research

This study is not free of limitations. First, we have to consider limitations coming from the way the variables were measured. Our evaluation of authentic leadership originated from employees and was aggregated as intersubjective at an entrepreneur-firm manager level (Level 2). Although such a technique is commonly used in research (e.g., [21]), collecting self-rated data on authentic leadership from entrepreneurs themselves would allow for an analysis of these relationships from a different perspective and for a comparison of the level of authenticity perceived by followers and leaders. On the other hand, evidence from past research shows that employees’ shared perceptions provide a more realistic view of their superiors’ authenticity [29]. Moreover, leadership is proposed to be an idea in followers’ minds, which is why discovering their perceptions is crucial [87,88]. Subsequently, the evaluation of employees’ individual innovative behaviour was based on self-reports. However, gathering such data from other sources does not guarantee a more objective measurement [33]. Future studies may try, however, use behavioural measures of the variables [36].

Second, our multilevel analyses allowed us to state that authentic leadership is a significant predictor of employee innovative behaviour as well as their personal initiative and work engagement, even though causal relationships cannot be confirmed. For future research, longitudinal, experimental, or quasi-experimental models are recommended to examine the consequences of authentic leadership, antecedents of employee innovation, and causal relationships between them. In longitudinal studies, attention should be paid to a time lag between measures of different constructs, especially when assessing leader behaviour and its outcomes [89].

Third, the design of the study involved examining employees from three European countries, which allowed us to generalize the results more broadly and test for culture effects. Nevertheless, more research on other cultural contexts is needed. Researchers may look not only at cultural dimensions [67] but also at other variables (e.g., power perception), which may be important for innovation [90,91]. Investigating cross cultural differences and effects of national policy, infrastructure, cultural values and practices would, however, require a sample of at least 30 countries to allow for multi-level (3 level) modelling [80,81].

We also did not investigate mechanisms responsible for the innovative behaviour of entrepreneur-firm owners, which may be different than those of employees [92]. This investigation awaits future studies.

### 4.2. Recommendations for Practice

Our results can be useful for managers seeking to encourage employee innovative behaviour, especially those of small firms. Entrepreneurs aiming at introducing innovative changes in their businesses, which originate from the innovative behaviour of employees, should pay attention to the leader-follower relationship. Approaching personnel with self-awareness, the balanced processing of information, relational transparency, and an internalized moral perspective translates into positive employee work attitudes such as higher innovative behaviour, taking more personal initiative and exhibiting higher work engagement. This study adds to previous research demonstrating that authentic leadership positively impacts well-being and the positive work attitudes of employees, which in turn affects performance [93] and effectiveness [94]. Consequently, one of the management strategies could be to emphasize building sincere relations with employees based on open communication so that supervisors are perceived as more authentic.

These findings suggest that the innovative behaviour of employees can be boosted through leadership training aimed at improving the quality of relationships between leaders and subordinates. Seeking and implementing innovative solutions may also be furthered by interventions aimed at strengthening employees’ personal initiative and work engagement.

## 5. Conclusions

To conclude, we proposed and tested a model explaining the relationships between authentic leadership and employees’ innovative behaviour. Our findings confirmed that small business owner–managers’ authentic leadership, as perceived by their employees, is related to innovative behaviour of their employees. We investigated two mechanisms mediating this relationship. Our results demonstrated that authentic leadership style predicts the personal initiative and work engagement of employees, and these two variables in turn predict employees’ innovative behaviour. We also detected a culture effect: being an employee from Spain (in comparison to Dutch and Polish employees) decreases the level of innovative behaviour demonstrated in daily activities in an organization. Our findings may be used to inform leaders about the organizational consequences of their leadership style.

## Figures and Tables

**Table 1:** Measurement invariance of authentic leadership, personal initiative, work engagement and innovative behaviour measures across samples of employees from the three countries.

Model	χ^2^	*df*	*p*	RMSEA	SRMR	CFI	Model Comparison	ΔRMSEA	ΔSRMR	ΔCFI
Authentic leadership										
M1. Configural invariance	565.324	177	<0.001	0.055	0.042	0.936	-	-	-	-
M2. Metric invariance	595.747	195	<0.001	0.053	0.044	0.934	M2 vs. M1	0.002	0.002	0.002
M 3. Scalar invariance	902.881	221	<0.001	0.065	0.116	0.887	M3 vs. M2	0.012	0.072	0.047
M 4. Partial scalar invariance	565.324	177	<0.001	0.064	0.116	0.893	M4 vs. M2	0.011	0.072	0.041
Personal initiative										
M1. Configural invariance	185.559	42	<0.001	0.069	0.050	0.934	-	-	-	-
M2. Metric invariance	218.633	54	<0.001	0.065	0.066	0.924	M2 vs. M1	0.004	0.016	0.010
M 3. Scalar invariance	450.180	68	<0.001	0.088	0.071	0.824	M3 vs. M2	0.023	0.005	0.100
M 4. Partial scalar invariance	185.559	42	<0.001	0.088	0.069	0.828	M4 vs. M2	0.023	0.003	0.096
Work engagement										
M1. Configural invariance	259.246	72	<0.001	0.060	0.040	0.957	-	-	-	-
M2. Metric invariance	314.451	84	<0.001	0.061	0.067	0.947	M2 vs. M1	0.001	0.027	0.010
M 3. Scalar invariance	648.429	102	<0.001	0.086	0.074	0.874	M3 vs. M2	0.019	0.007	0.073
M 4. Partial scalar invariance	259.246	72	<0.001	0.085	0.075	0.880	M4 vs. M2	0.024	0.008	0.067
Innovative behaviour										
M1. Configural invariance	43.56	18	0.001	0.045	0.028	0.992	-	-	-	-
M2. Metric invariance	68.24	28	0.001	0.045	0.048	0.987	M2 vs. M1	0.000	0.021	0.005
M3. Scalar invariance	76.269	30	0.001	0.047	0.062	0.985	M3 vs. M2	0.002	0.013	0.002

*Note.* RMSEA = Root Mean Square Error of Approximation; SRMR = Standardized Root Mean Square Residual; CFI = Comparative Fit Index. To evaluate the model fit, we applied the recommended model fit criteria: for RMSEA and SRMR values below 0.08 indicate acceptable fit, and values above 0.10 indicate poor fit; for CFI, values higher than 0.90 show an acceptable model fit [77,78].

**Table 2:** Descriptive statistics and correlations between the study variables among employees from the three countries.

Variable	*M*	*SD*	1	2	3	4
1. Authentic leadership	32.75	10.48	-	0.20	0.30 **	0.35 **
2. Personal initiative	26.02	4.79	0.23 **	-	0.70 **	0.62 **
3. Work engagement	34.60	9.55	0.37 **	0.53 **	-	0.61 **
4. Innovative behaviour	18.75	4.92	0.28 **	0.53 **	0.48 **	-

*Note*. ** *p* < 0.01 (two tailed); lower diagonal are the de-aggregated data (711 employees), upper diagonal are aggregated data (85 organizations).

**Table 3:** Results of hierarchical multilevel modelling explaining employee innovative behaviour.

Predictor	Model 1			Model 2			Model 3			Model 4		
	γ	SE	*p*	γ	SE	*p*	γ	SE	*p*	γ	SE	*p*
Country	–2.02	0.63	0.002	–2.03	0.64	0.002	–2.03	0.64	0.002	–2.04	0.64	0.002
Authentic leadership	1.09	0.31	0.001	1.07	0.32	0.001	1.09	0.31	0.001	1.07	0.32	0.001
Personal initiative	-	-	-	0.47	0.05	0.001	-	-	-	0.38	0.05	0.001
Work engagement	-	-	-	-	-	-	0.23	0.03	0.001	0.13	0.03	0.001
Level 1 variance	17.58			13.14			14.21			12.37		
Level 2 variance	5.35			6.01			5.77			6.11		

*Note*. γ = unstandardized gamma coefficient, SE = standard error.
